# Supplementary material for: The genome sequence of a braconid wasp, Dinocampus coccinellae (Schrank, 1802)
Source: Wellcome Open Res. 2024 Aug 12;9:461. [Version 1] doi: 10.12688/wellcomeopenres.22862.1 (PMC11464979; doi:10.12688/wellcomeopenres.22862.1)
Supplement: Peer review history

# Reviewer report: Reviewer response for version 1

**DOI:** 10.21956/wellcomeopenres.25175.r96242

**Published:** 2024-10-09

**Authors:** Zijun Xiong

**Affiliations:** 1 BGI-Research, Wuhan, China

The manuscript presents a chromosome-level assembly of the braconid wasp, *Dinocampus coccinellae*, utilizing PacBio long-read sequencing technology and Hi-C technology. The assembled genome has a scaffold N50 of 17.9 Mb, a total genome size of 110.4 Mb, and is anchored into 8 chromosomes.

 I have several issues that I would like the authors to address:

1. As the authors mentioned, a previous study (Sethuraman et al., 2022) has already published the genome of *Dinocampus coccinellae*. When comparing this published genome, the new chromosome-level assembly shows a reduction of 52 Mb in genomic sequences, accounting for 47% of the new genome (162.3 Mb vs. 110.4 Mb). What regions are missing? Are they all composed of anonymous bases? Why is there such a significant difference in genome size? It appears that the authors used a genome size of 120 Mb to calculate the genome coverage (177-fold). Why do you think the genome size of *Dinocampus* is 120 Mb? Have you estimated the genome size of this species?
2. The authors referred to the genome assembly as ‘pseudomolecules’ in the abstract and Table 3. I think it is incorrect to use the term “pseudo.” Hi-C scaffolding with YaHS enabled the construction of physical chromosome-scale scaffolds. Can you explain why you used ‘pseudo’ to describe the chromosomes?
3. The complete BUSCO score is only 94.6%, which is lower than the benchmark and also lower than other published Hymenoptera genomes, such as those of bees, wasps, and ants (PMID: 33319913, 37164995). Although HiFi sequencing reads are of high accuracy, I suggest using high-quality short reads to further polish the genome.
4. Figures 3 and 4 indicate that the genome contains 10 scaffolds, but the number of scaffolds is listed as 9 in Tables 2 and 3. What is the 10th scaffold? In Figure 4, does the x-axis represent the scaffold number? Why are all 10 scaffolds shown with the same length?
5. The size of the mitogenome is 22.88 kb, which is higher than the typical insect mitochondrial genome (14–19 kb in length, PMID: 34887433). Can you confirm that the mitogenome length is accurate?

Additionally, I suggest adding a length scale to Figure 1.

Are sufficient details of methods and materials provided to allow replication by others?

Yes

Is the rationale for creating the dataset(s) clearly described?

Yes

Are the datasets clearly presented in a useable and accessible format?

Yes

Are the protocols appropriate and is the work technically sound?

Yes

Reviewer Expertise:

Phylogeny, Comparative genomics, Insect evolution

I confirm that I have read this submission and believe that I have an appropriate level of expertise to confirm that it is of an acceptable scientific standard.

# Reviewer report: Reviewer response for version 1

**DOI:** 10.21956/wellcomeopenres.25175.r97341

**Published:** 2024-09-18

**Authors:** Avinjikkattu Parambil Ranjith

**Affiliations:** 1 Integrative Insect Ecology Research Unit, Chulalongkorn University, Bangkok, Bangkok, Thailand

I have reviewed the manuscript 'The genome sequence of a braconid wasp, *Dinocampus coccinellae *(Schrank, 1802)' and added a few comments.

 The manuscript offers an important contribution to the genome wise study on the braconid parasitoid wasps, euphorines in particular. 

 I have checked the taxonomic part mostly and found some areas to modify/improve.

- Keywords
- Please arrange keywords in alphabetical order
- In keywords
- Please italicize the species name of the parasitoid
- Line 3 in Introduction
- Please change ladybird to ladybird beetles
- Line 9 in Introduction
- Please add one citation for the sentence to support the statement (Maure et al. 2011)
  Maure F, Brodeur J, Ponlet N, Doyon J, Firlej A, Elguero1 E,Thomas F (2011) The cost of a bodyguard. Biology Letters,7, 843-846.

Are sufficient details of methods and materials provided to allow replication by others?

Yes

Is the rationale for creating the dataset(s) clearly described?

Yes

Are the datasets clearly presented in a useable and accessible format?

Yes

Are the protocols appropriate and is the work technically sound?

Yes

Reviewer Expertise:

My area of research mainly lies in the taxonomy, systematics and ecology of hymenopteran parasitoids.

I confirm that I have read this submission and believe that I have an appropriate level of expertise to confirm that it is of an acceptable scientific standard.

# Reviewer report: Reviewer response for version 1

**DOI:** 10.21956/wellcomeopenres.25175.r97334

**Published:** 2024-09-11

**Authors:** Christophe Klopp

**Affiliations:** 1 INRAE, Castanet-Tolosan, France

The authors present a novel assembly of the *Dinocampus coccinellae *genome. The article is technically sound and well written.

 The authors have followed a four-step procedure beginning with the HiFi read assembly with hifiasm, then the produced contigs have been purged with purge_dups, next scaffolded with Hi-C using YaHS and last, they have manually curated the generated scaffolds with PretextView.

 They do not seem to have included the Hi-C reads in the assembly phase which is recommended by hifiasm developers to improve contig phasing. Therefore, the contig have a higher risk to be haplotype chimeras. They have also chosen to use the --primary option which produces primary and alternative contigs sets, the alternative contigs set only containing contigs which are different from the primary and this makes it quite difficult to use in following analysis. A better option would have been to produce hap1 and hap2 which correspond to two contigs sets completely covering genome which can both be scaffolded in chromosomes.

Are sufficient details of methods and materials provided to allow replication by others?

Yes

Is the rationale for creating the dataset(s) clearly described?

Partly

Are the datasets clearly presented in a useable and accessible format?

Yes

Are the protocols appropriate and is the work technically sound?

Yes

Reviewer Expertise:

bioinformatics, genome assembly and annotation

I confirm that I have read this submission and believe that I have an appropriate level of expertise to confirm that it is of an acceptable scientific standard, however I have significant reservations, as outlined above.
